# Voiding Behavior and Efferent Bladder Function Altered in Mice Following Social Defeat but Not Witness Trauma

**DOI:** 10.3389/fphys.2020.00247

**Published:** 2020-03-20

**Authors:** Eliza G. West, Donna J. Sellers, Russ Chess-Williams, Catherine McDermott

**Affiliations:** Centre for Urology Research, Faculty of Health Sciences and Medicine, Bond University, Robina, QLD, Australia

**Keywords:** bladder, psychological stress, social defeat, witness trauma, urinary retention, voiding behavior

## Abstract

Psychological stress is associated with bladder dysfunction, however, the local bladder mechanisms affected are not well understood. This study aimed to determine how psychological stress, caused by social defeat or witness trauma, affects voiding behavior and bladder function. Pairs of male C57Bl/6J mice were placed in a custom-made plexiglass chamber with an aggressor ARC(S) mouse for 1 h/day for 10 days. The social defeat mouse was in physical contact with the aggressor, while the witness was physically separated but could observe interactions between its cage-mate and the aggressor. Age matched control pairs were used for comparison. Voiding analysis was conducted periodically over the 10 days. An *ex vivo* whole bladder preparation was used to assess functional changes after the period of stress. Plasma corticosterone levels were significantly increased by both social defeat and witness trauma stress when compared to unstressed controls. Voiding analysis revealed a significant decrease in voiding frequency in the social defeat group compared to control animals, indicating an altered voiding phenotype. Witness trauma did not alter voiding behavior. Bladder contractile responses to cholinergic stimulation were not significantly altered in either stress group, nor was relaxation to the beta-adrenoceptor agonist isoprenaline. However, nerve evoked contractile responses were significantly increased at all frequencies in bladders from social defeat but not witness trauma mice. Purinergic contractile responses were also significantly enhanced in this group. Social defeat also resulted in increased urothelial acetylcholine release during bladder distension, with no change in ATP release. In conclusion, functional bladder changes are dependent upon stressor type. Enhanced urothelial acetylcholine may desensitize bladder sensory nerves, which, coupled with more efficient voiding contractions due to enhanced nerve-mediated and purinergic detrusor responses, may account for the altered voiding phenotype observed. This study reports a male model of social defeat stress with reduced urinary frequency, with no voiding changes observed in the witness.

## Introduction

Psychological stress is known to affect a large portion of society. It cannot be defined by any one cause and even depends on a person’s surroundings. Stressors, no matter what severity, have been documented to promote behavioral and physiological disturbances (Schneiderman et al., 2005). It has been documented that psychological stress can impact several visceral functions with pathological consequences. Previously, stress has been linked to structural changes in the brain (Sapolsky et al., 1990), changes to the immune system (Stephens and Gary, 2012) and dysfunction in the gastrointestinal and cardiovascular systems (Qin et al., 2014). Bladder dysfunction, such as overactive bladder (OAB), incontinence and interstitial cystitis are common in the general population (Teleman et al., 2004) and a body of clinical evidence exists linking bladder disorders with stress, anxiety and depression, including witness trauma and post-traumatic stress disorder (Rothrock et al., 2001; Lai et al., 2015, 2016; Bradley et al., 2017).

Several observational clinical studies have investigated the general correlation between bladder pathologies and psychological stress. Stress appears to greatly influence the development of bladder symptoms (Bradley et al., 2014) or worsens symptom severity (Lai et al., 2015). OAB and interstitial cystitis patients report equivalent high psychological stress levels, which are significantly higher than healthy controls (Lai et al., 2015). OAB has been linked to traumatic events in childhood or adult life, with OAB occurring in 22% of deployed female veterans (Lai et al., 2015; Bradley et al., 2017) and a high proportion of adult OAB patients have reported experiencing childhood sexual trauma (Lai et al., 2016). Higher levels of psychological stress are also related to worsening of symptoms, with greater pain and urinary urgency in patients with interstitial cystitis (Rothrock et al., 2001). While this information gives an indication of the impact of psychological stress on the bladder, the mechanisms underlying this dysfunction remain unclear.

Some research groups have attempted to fill this knowledge gap using different rodent models of psychological stress, including water avoidance (Smith et al., 2011; Lee et al., 2015), immobilization stress (Spanos et al., 1997) and social defeat (Chang et al., 2009; Wood et al., 2009). Water avoidance stress caused increased urinary frequency in female rats, which was associated with increased infiltration and activation of mast cells and angiogenesis in the bladder (Smith et al., 2011). In contrast, social defeat in male rodents has been reported to alter voiding differently, with less frequent voiding events observed in mice exposed to social defeat (Chang et al., 2009) and one single large void typically observed (Mann et al., 2015). This altered voiding behavior in social defeat mice and rats is referred to in the literature as a urinary retention phenotype and is associated with bladder wall hypertrophy, urodynamic changes including increased bladder capacity and micturition volume, and upregulation of cortico-tropin releasing factor (CRF) in Barrington’s nucleus neurons (Wood et al., 2009). While these studies have been successful in assessing changes in voiding behavior, the precise changes in local bladder physiology and the underlying mechanisms associated with stress-induced bladder dysfunction are not fully understood.

Local afferent and efferent systems contribute to maintaining normal bladder function. During bladder filling, sympathetic activity relaxes the detrusor muscle via β-adrenoceptor stimulation, limiting the increase in intravesical pressure. During voiding, the parasympathetic system initiates detrusor contraction by co-release of acetylcholine (ACh) and ATP which act via M3 muscarinic receptors and P2 × 1 purinoceptors respectively (Sellers et al., 2000; Burnstock, 2013). The urothelium which lines the bladder lumen plays an important signaling role, releasing chemical mediators during the filling phase (Merrill et al., 2016). These mediators which include ATP and ACh, have been reported to act on sensory nerve fibers in the sub-urothelium, the detrusor muscle, as well as having autocrine actions on the urothelium (Birder et al., 2012). Changes in urothelial mediator release are implicated in bladder dysfunction (Kumar et al., 2007; Birder et al., 2012). To date the impact of psychological stress on these local bladder mechanisms has not been investigated.

Social stress is a widespread environmental factor that can affect health and research has shown that experiencing or witnessing traumatic events can increase the risk of anxiety, depression and PTSD (Patki et al., 2015). In the traditional rodent models of physical social stress, the subordination of one male by a larger and more aggressive male simulates a socially-induced psychological stressor and leads to depression and anxiety-like behaviors (Golden et al., 2011). Recent experimental studies demonstrate a new rodent model that better distinguishes between physical and emotional social stress (Sial et al., 2016; Li et al., 2018). Social defeat and witness trauma stress replicates acute social physical and emotional stress respectively, and these stresses, when repeated over time, stimulate chronic stress.

While the social defeat model has previously been used to investigate changes in bladder function, the effect of witness trauma on the bladder has not yet been assessed. It is important to consider the impact of psychological stress on bladder function beyond the effects on voiding, and to understand the physiological mechanisms underlying the bladder dysfunction that are altered by psychological stress. Therefore, the aim of this study was to investigate the effects of social defeat and witness trauma on voiding behavior in mice and investigate accompanying changes in local bladder function including bladder compliance, contractile responses and urothelial mediator release.

## Materials and Methods

### Animal Model

All experimental procedures were performed in accordance with the Australian Code for the Care and Use of Animals for Scientific Purposes and with the approval of the University of Queensland Animal Ethics Committee. Adult male C57BL/6JArc mice (12–14 weeks in age) were used for this study and housed under environmentally controlled conditions, with 12 h light-dark cycles and access to food and water *ab libitum*. Animals were randomly allocated into three experimental groups: Control, Social Defeat, and Witness.

### Social Defeat/Witness Trauma Model

A model of physical (social defeat) and emotional (witness trauma) social stress was employed using a variation of the protocols previously described by Sial et al. (2016) and Li et al. (2018). Male ex-breeder ARC(S) mice were screened for persistent aggressive behavior and housed individually. Aggressor mice we used in rotation, so social defeat mice did not encounter the same aggressor on consecutive days. Male C57Bl/6J mice (12–14 weeks) were housed in pairs for 3 days prior to and during the 10 days stress protocol, each being randomly allocated to either the (1) social defeat or (2) witness trauma experimental group. C57Bl/6J pairs were placed in a custom-made plexiglass chamber with the aggressor mouse for 1 h/day for 10 days. The social defeat mouse was in physical contact with the aggressor for a maximum of 5 min and then separated by a transparent perforated barrier for 55 min. To minimize risk of wounding, all social defeat sessions were observed continuously, and the animals separated if the social defeat mouse exhibited clear submissive behavior, including submissive posture or freezing. Wounding but not superficial scratches is associated with innate immune response (Foertsch et al., 2017) and through observed defeat sessions no social defeat animals received wounds requiring their exclusion from the experiment. The witness mouse was physically separated from the other mice during the stress protocol by a transparent perforated wall but could observe interactions between its cage mate and the aggressor. Age matched control pairs were housed under normal conditions for the duration of the study.

### Voiding Pattern Analysis

Voiding analysis was used to assess changes in voiding behavior and was performed as previously described (West et al., 2018) before the first stress protocol (day 0) and repeated on days 1, 3, 7, and 10 of the stress protocol, before euthanasia. All voiding pattern analysis was performed in the morning, beginning at the change-over of the light/dark cycle. Mice were housed singly in cages lined with Filtech^®^ Hardened Ashless Filter Paper #225 for 4 h with free access to food and water. The filter paper was collected, and urine spots detected using a Molecular Imager ChemiDoc XRS ultraviolet transilluminator (#720BR1293 BioRad, CA, United States). The papers were photographed, digitized, and analyzed using Image J software.

### Isolated Whole Bladder Preparation

Mice were euthanized by cervical dislocation 24 h after the 10th daily stress exposure. A venous blood sample was taken at the time of euthanasia, and plasma corticosterone levels quantified using the Corticosterone Competitive ELISA (Invitrogen) according to the manufacturer’s instructions. Blood samples were collected in the morning to avoid variation due to diurnal changes in corticosterone levels. The bladder was then isolated and a three-way cannula inserted via the urethra, to enable recording of intravesical pressure in addition to bladder filling and emptying as previously described (West et al., 2018), and mounted into a modified tissue bath (8 mL), containing gassed (95% O_2_/5% CO_2_) Krebs bicarbonate solution (composition in mM: NaCl 118, NaHCO_3_ 24.9, CaCl_2_ 1.9, MgSO_4_ 1.15, KCl 4.7, KH_2_PO_4_ 1.15, and D-glucose 11.7) at 37°C. Intravesical pressure was measured using a pressure transducer (GlobalTown^®^ Microtech) connected to a PC via a PowerLab data acquisition system (AD Instruments), using LabChart 7 software (AD Instruments).

Following equilibration for 30 min, bladder distensions were performed by intravesical infusion of isotonic saline at a rate of 30 μL/min up to a luminal pressure of 40 mmHg to check viability and compliance, with all further distensions for experimental purposes to 20 mmHg. The urothelium is known to play a signaling role, releasing several chemical mediators in response to distension during bladder filling. Following distension to 20 mmHg the bladder was drained via the two-way cannula and intraluminal contents collected for measurement of the urothelial mediators ATP and ACh. Samples were immediately frozen on dry ice and stored at −80°C for later assay of ATP and ACh, using the ATP Determination Kit and Acetylcholine Amplex(^®^) Red Assay Kit (Molecular Probes). The assay was performed according to manufacturer instructions and luminescence or fluorescence (Ex. 540/Em. 590 nm) measured, using a Modulus micro-plate reader (Promega).

Following distension to 20 mmHg, bladders were allowed to equilibrate and electrical field stimulation (EFS) was undertaken. The bladders were electrically stimulated using 5 s trains of pulses (50V, 0.5 ms pulse width), delivered every 100 s at 1, 5, 10, and 20 Hz and contractions were measured as pressure change from baseline. EFS was repeated at 20 Hz in the presence of L-NNA (100 μM), followed by addition of atropine (1 μM) and finally αβ-methylene ATP (10 μM), all of which were added to the serosal solution. Neurogenic origins of the pressure response to EFS were confirmed using tetrodotoxin (0.1 μM), which abolished responses at all frequencies.

Pressure responses to pharmacological agents was also assessed by serosal addition of αβ-methylene-ATP (10 μM), cumulative carbachol concentrations, and relaxation to cumulative isoprenaline concentrations. Finally, 60 mM KCl solution was added, to measure non-receptor mediated contractile response in the whole bladder. All contractions and relaxation responses were measured as a change in pressure from baseline. The maximum duration of whole bladder preparation experiments was 4 h.

### Data and Statistical Analysis

All experiments were randomized, with 6 mice per experimental group and each experimental protocol started on a different day. Results are expressed as mean ± standard error of the mean (SEM). Data were analyzed using ordinary one-ANOVA with Dunnett multiple comparisons test or repeated measures two-way ANOVA with Bonferroni’s multiple comparisons test, using GraphPad Prism version 6 software (GraphPad, San Diego, CA, United States). Significance levels were defined as *P* < 0.05 (^∗^).

## Results

### Effects of Psychological Stress on Animal Parameters and Voiding Behavior

**FIGURE 1:**
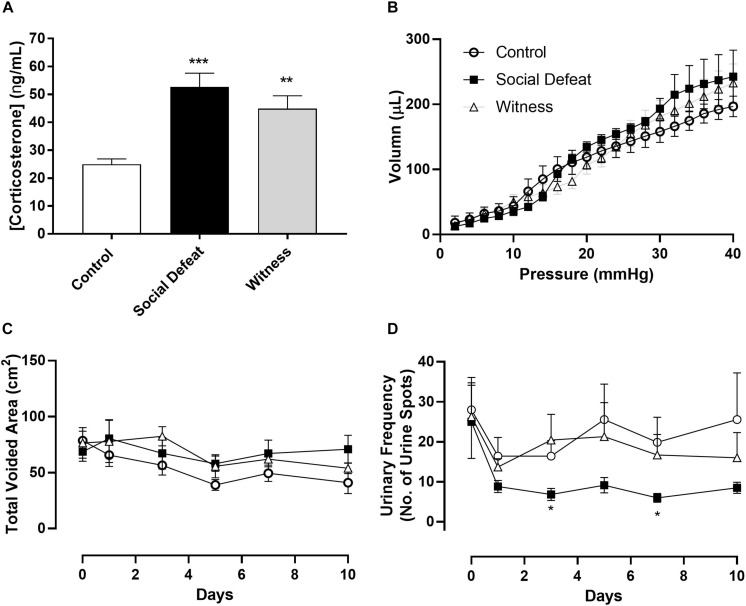
**(A)** Plasma corticosterone levels, **(B)** bladder volume-pressure relationship, **(C)** total voided area, and **(D)** urinary frequency (no. of voids over 4 h period) in control, social defeat and witness mice. Data represents mean ± S.E.M. (*n* = 6) and was analyzed using one-way ANOVA **(A)** or two-way ANOVA with Bonferroni multiple comparisons test **(A–D)** (*p < 0.05, **p < 0.01, and ***p < 0.001 vs. control).

Animal body weight and water consumption were measured on days 0 (baseline data – Table 1) and on 1, 3, 5, 7, and 10 days following first stress exposure. These parameters were unchanged throughout the stress protocol with neither the social defeat nor witness trauma significantly affecting body weight or water consumption (data not shown). There was no significant difference in bladder weight between the control, social defeat or witness groups (Table 1). A blood sample was taken at the time of euthanasia for analysis of plasma corticosterone. There was a significant increase in plasma corticosterone from 24.9 ± 1.93 ng/mL in control mice to 54.7 ± 4.87 ng/mL in social defeat (*p* = 0.0001) and 44.86 ± 4.66 ng/mL in witness mice (*p* = 0.0037) (Figure 1A), indicating that a hormonal stress response was present in both stress groups.

**TABLE 1:** Baseline animal parameters and whole bladder responses to carbachol and isoprenaline (% of pre-contraction) in control, social defeat and witness mice.

	Control	Social defeat	Witness
**Animal Parameters**			
Body weight (g)	26.3 ± 0.67	26.7 ± 0.68	26.3 ± 1.04
Bladder weight (mg)	22.3 ± 1.18	21.5 ± 0.49	22.96 ± 0.41
Water consumption (g)	0.64 ± 0.19	0.79 ± 0.23	0.98 ± 0.66
**Whole Bladder Responses**			
Carbachol			
pEC_50_	6.19 ± 0.08	6.04 ± 0.10	6.01 ± 0.09
Maximal response (mmHg)	44.0 ± 2.19	39.08 ± 2.36	45.35 ± 2.97
**Isoprenaline**			
pIC_50_	6.65 ± 0.18	6.73 ± 0.31	6.65 ± 0.17
Maximal response (% of pre-contraction remaining)	−4.82 ± 6.78	11.8 ± 8.88	7.13 ± 6.24

**Data is presented as mean ± S.E.M. (n = 6) analyzed using one-way ANOVA with Dunnett multiple comparisons test.**

Voiding behavior was assessed in all three experimental groups. There was no significant change in total voided urine over time or between stress and control groups, indicating that the rate of urine production was not affected by 10 days social defeat or witness trauma (Figure 1C). However, social defeat resulted in a significant decrease in urinary frequency (Figure 1D), with a significant change evident following 3 (*p* = 0.027) and 7 days (*p* = 0.019) stress exposure, a change that was not observed in the witness group. The absence of changes in water consumption and total voided volume indicate that this an actual change in urinary frequency and does not reflect reduced urine production. Effects of psychological stress on bladder compliance and contractile responses.

**FIGURE 2:**
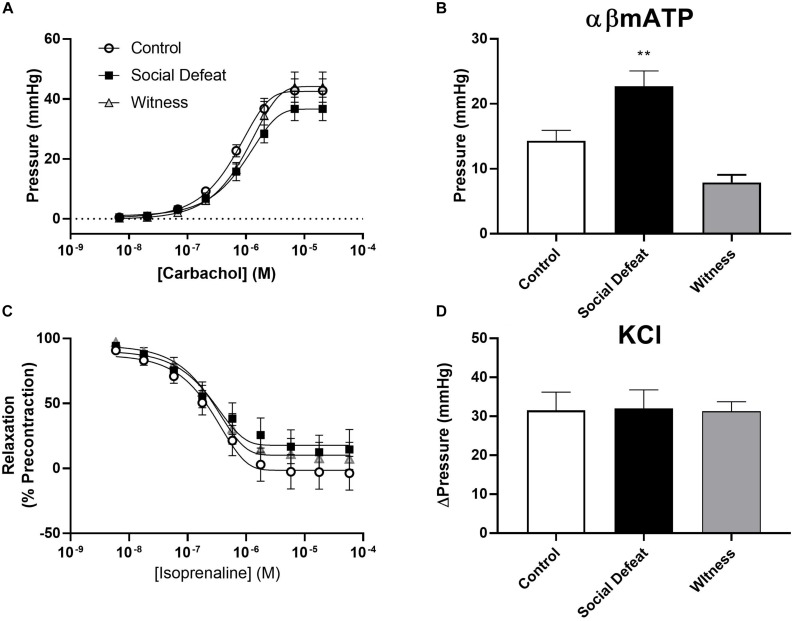
Isolated whole bladder responses to **(A)** the muscarinic agonist carbachol, **(B)** the purinergic agonist αβ-mATP (10 μM), **(C)** the beta-adrenoceptor agonist isoprenaline and **(D)** KCl (60 mM). Data represents mean ± S.E.M. (*n* = 6) and was analyzed using non-linear regression curve fit analysis **(A,C)** or one-way ANOVA with Dunnett’s multiple comparisons test **(B,D)** (**p < 0.01 vs. control).

Bladder compliance was not significantly altered in social defeat or witness mice when compared to controls, with no change in volume-pressure relationships during bladder filling observed (Figure 1B). A concentration dependent increase in intravesical pressure was observed upon addition of carbachol to bladders from all groups (Figure 2A). This response was not significantly affected by stress (Figure 2A and Table 1), nor was the contractile response to KCl (Figure 2D). However, stimulation of purinergic receptors with αβ-methylene-ATP (10 μM) produced a significantly greater pressure response in bladders from social defeat mice compared to control (*p* = 0.007) (Figure 2B). This change was not evident in the witness group. Relaxation to isoprenaline following carbachol pre-contraction was not significantly affected by social defeat or witness stress with no change in maximal response or pIC_50_ evident (Figure 2C and Table 1).

**FIGURE 3:**
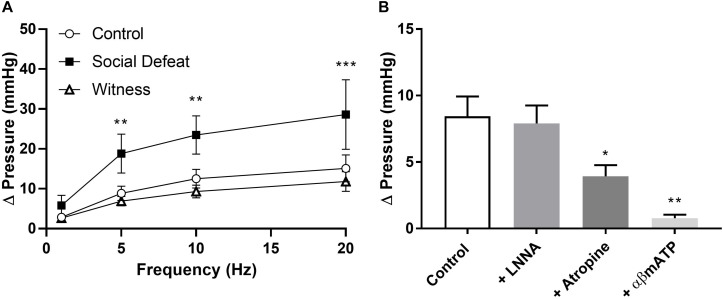
**(A)** Nerve-evoked pressure response of isolated whole bladders from control, social defeat and witness mice at 1, 5, 10, and 20 Hz. **(B)** Nerve-mediated contractions to EFS at 20 Hz in isolated whole bladders from control mice in the presence of L-NNA (100 μM), atropine (1 μM) and α,β-methylene-ATP (10 μM). Responses were recorded as change in pressure from baseline. Data are presented as mean ± S.E.M. (*n* = 6). Data analyzed using **(A)** two-way ANOVA with Bonferroni multiple comparisons test or **(B)** one-way ANOVA with Dunnett multiple comparisons test (*p < 0.05, **p < 0.01, ***p < 0.001 vs. control).

Electric field stimulation (EFS) was used to determine if social defeat or witness trauma affected efferent nerve-mediate bladder contraction. EFS produced a frequency-dependent increase in intravesical pressure in all bladders, with the response to EFS being significantly enhanced at 5, 10, and 20 Hz (*p* = 0.004, *p* = 0.001, *p* = 0.0001 respectively) in the social defeat group when compared to control (Figure 3A). However, witness trauma did not change responses to EFS. To determine the relative contribution of ACh and ATP to nerve evoked contractions, muscarinic and purinergic receptors were antagonized and desensitized using atropine and αβ-mATP respectively. Atropine significantly reduced the response to EFS by 45.9 ± 5.77% in bladders from control mice (*p* = 0.023) (Figure 3B), with a significantly smaller muscarinic component evident in the social defeat group, with a decrease of 22.7 ± 5.61% observed (*p* = 0.033). Desensitization of purinergic receptors produced a further reduction in the nerve-evoked bladder response in all groups, and this was greatest in bladders from social defeat mice (57.2 ± 8.93%) compared to in controls (39.8 ± 6.66%) (Figure 3B). The muscarinic and purinergic contributions to nerve-mediated contraction were similar in the witness and control groups (data not shown). The presence of the nitric oxide synthase inhibitor L-NNA (100 μM) did not alter responses to EFS in any of the experimental groups.

### Effects of Psychological Stress on Intraluminal Release of ATP and ACh

**FIGURE 4:**
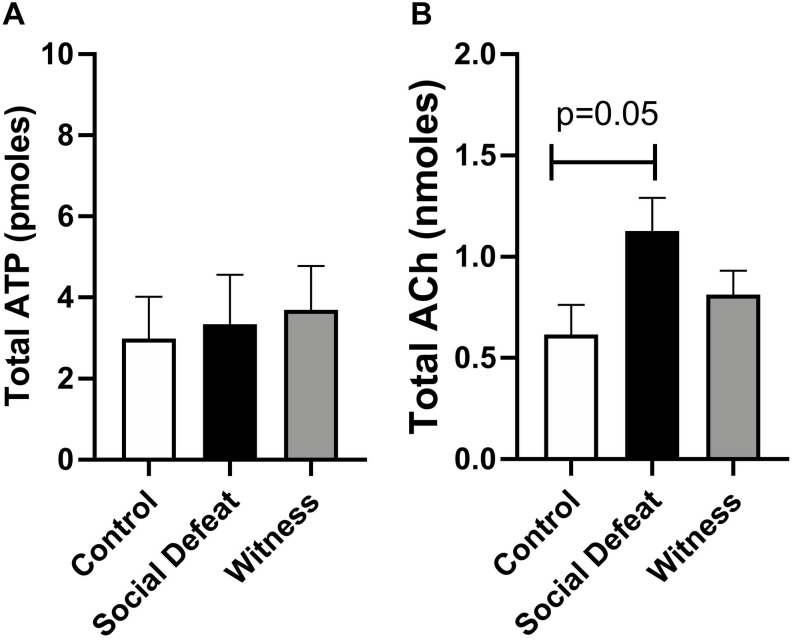
Intraluminal release of **(A)** ATP and **(B)** ACh from isolated bladders from control, social defeat and witness mice in response to distension to 20 mmHg. Data are presented as mean ± S.E.M. (*n* ≥ 5) and were analyzed using one-way ANOVA with Dunnett multiple comparisons test.

Samples of intraluminal fluid were collected from isolated whole bladders distended to 20 mmHg and analyzed to quantify total release of the urothelial mediators ATP and Ach to determine if changes in their release contributed to stress induced bladder dysfunction. Total ACh release was greater than total ATP release in all groups (Figure 4), which is consistent with previous reports (West et al., 2018). Total intraluminal ATP was similar in all experimental groups and was not affected by either stress exposure (Figure 4A). However, total release of ACh into the bladder lumen was enhanced in the social defeat group (1.13 ± 0.16 nmoles) when compared to controls (0.73 ± 0.17 nmoles), although this change did not meet statistical significance (*p* = 0.05) (Figure 4B).

## Discussion

There is abundant clinical evidence that psychological stress affects bladder function and is associated with bladder pathologies. Currently, there is a lack of understanding of the physiological changes within the bladder due to ongoing psychological stress. This study employed a novel paired animal model of physical and emotional social stress to determine if social defeat and witness trauma impact the bladder in the same way.

Exposure to physical or emotional stress produced a similar hormonal stress response in both groups, confirming that the mice used in this study were stressed when compared to controls. This is consistent with other experimental studies which assessed corticosterone levels in the social defeat model (Mann et al., 2015), as well as paired social defeat/witness trauma model (Sial et al., 2016; Li et al., 2018). Interestingly, while social defeat resulted in reduced urinary frequency, which supports previous reports of voiding dysfunction in this model (Chang et al., 2009; Mann et al., 2015), voiding was not altered in witness trauma mice despite a similar hormonal stress response. Mann et al. (2015) similarly found that like witness trauma, chronic restraint stress did not alter bladder function, with similar voiding, bladder mass and bladder wall thickness compared to controls. A recent study, that explored how social support affects stress-induced changes in social behavior produced by the social defeat/witness trauma paradigm used here, found that the social support provided by paired housing following stress exposure had protective effects in the witness but not in social defeat mice (Li et al., 2018). This may explain why despite similar changes in plasma corticosterone, no voiding changes were evident in the witness group. The benefits of social support reported by Li et al. (2018) was however in adolescent mice; this as yet has not been assessed in adult mice like those used in the present study and is worthy of future investigation.

In addition to stressor-specific differences seen in the present study, sex differences in psychological stress-induced bladder dysfunction and brain responses have previously been reported. A recent study identified consistent sex-specific differences in the brain responses to trauma in male and female rats using two models of post-traumatic stress disorder (Pooley et al., 2018). We found a significant increase in voiding frequency in female mice following water avoidance stress (West et al., 2019), an effect that has also been reported in female rats (Smith et al., 2011). however McGonagle et al. (2012) reported a decrease in voiding frequency in male mice following water avoidance stress, similar to the changes observed in the current study in male mice following social defeat.

To investigate the local bladder mechanisms that may contribute to the altered voiding phenotype in social defeat mice, bladder compliance, contractile response and release of urothelial signaling mediators were assessed using an *ex vivo* whole-bladder preparation. Contractile responses to muscarinic (carbachol) and receptor-independent (KCl) detrusor stimulation, as well as beta-adrenoceptor mediated bladder relaxation were not affected by stress. However, stimulation of purinergic receptors with αβmethylene-ATP produced a significantly greater pressure response in bladders from social defeat mice, suggesting that changes to the purinergic system may contribute to the altered voiding behavior observed. Similarly, nerve mediated bladder responses to EFS were increased at all frequencies in the social defeat group. This indicates that with social defeat stress, an increased intravesical pressure generated during voiding contractions likely contributed to the larger void size observed in social defeat mice. The reason for this is not entirely clear, however, from the response to αβmethylene-ATP we see that there is an enhancement of the purinergic component of the bladder. Research has shown that within the lower urinary tract of rodents there is co-transmission of ACh and ATP from efferent nerves (Burnstock, 2013). As there was no change to the cholinergic responses of the bladder, and EFS results indicate that the contribution of ACh to nerve-evoked contraction is significantly decreased, it appears that the upregulation of the purinergic component plays a role in bladder changes caused by social defeat. Purinergic signaling is involved in a number of physiological and pathophysiological events of the lower urinary tract, and research has shown that purinergic responses are often increased in diseased states such as interstitial cystitis and bladder pain syndrome (Knight et al., 2002; Burnstock, 2013).

Release of signaling molecules from the urothelium plays an important role in normal bladder function, and changes in release have been linked to lower urinary tract dysfunction (Merrill et al., 2016). As the bladder fills, stretch of the urothelium elicits release of ATP and ACh. ATP acts on purinergic receptors on low threshold sensory fibers in the suburothelium to initiate the micturition reflex and can give rise to perceptions of pain in bladder pathologies (Kumar et al., 2007). The role of urothelial Ach is still being identified, but it has been reported in influence sensory nerve activity, stimulate release of urothelial derived inhibitory factor to inhibit detrusor contraction, and stimulate urothelial contraction and pacemaker activity (Hawthorn et al., 2000; Templeman et al., 2002; Akino et al., 2008; Daly et al., 2010; Moro et al., 2011). While ATP levels were unchanged in the present study, release of ACh into the bladder lumen during filling was 55% greater in social defeat animals compared to controls. Although the role of urothelial ACh is not yet fully understood, stimulation of muscarinic receptors in bladder sensory nerves has been reported to depress sensory transduction (Daly et al., 2010). When we consider that bladder compliance was unchanged in social defeat mice, depression of sensory afferent fibers by enhanced urothelial ACh may explain the decrease in urinary frequency that occurs with social defeat, allowing a larger urine volume to be accommodated before afferent nerves are activated.

While the current study focused on changes to local bladder mechanisms involved in the control of bladder filling and voiding, other research has previously investigated the impact of social defeat on the pontine micturition center, also referred to as Barrington’s nucleus, which regulates the micturition reflex (Wood et al., 2009, 2013). The stress-related neuropeptide corticotropin-releasing factor (CRF) has been shown to be present in spinal projections of Barrington’s nucleus and is an inhibitory neuromodulator of the micturition pathway (Pavcovich and Valentino, 1995). Social defeat stress has been reported to increase the number of CRF immunoreactive neurons in Barrington’s nucleus (Wood et al., 2009), with administration of a CRF1 antagonist to social defeat mice restoring urodynamic function without affecting CRF expression in the control center (Wood et al., 2013). CRF is also distributed in visceral organs including the bladder, with serum and bladder CRF expression also increased by psychological stress in rats (Seki et al., 2019). This suggests that central, as well as local bladder changes, contribute to alterations in voiding behavior with social defeat.

The results presented here indicate that psychological stress affects bladder function, but this effect is dependent on stressor type. A decrease in bladder afferent sensitivity due to enhanced urothelial ACh release, coupled with more efficient nerve-evoked voiding contractions, due to enhanced purinergic responses, may contribute to the decrease in urinary frequency and increased void size observed here in social defeat mice. In conclusion, stress induced functional bladder changes are dependent on stressor type, with no voiding changes observed in the witness trauma model.

## Data Availability Statement

The datasets generated for this study are available on request to the corresponding author.

## Ethics Statement

The animal study was reviewed and approved by the University of Queensland Animal Ethics Committee.

## Author Contributions

DS, RC-W, and CM contributed to conception and design of the study, interpretation of results, and manuscript revisions. EW was responsible for data acquisition, analysis, and interpretation, and drafting the manuscript. EW and CM were responsible for statistical analysis. All authors contributed to manuscript revision, read and approved the submitted version.

## Conflict of Interest

The authors declare that the research was conducted in the absence of any commercial or financial relationships that could be construed as a potential conflict of interest.
